# Integrated treatment approach to Miller Fisher syndrome, a variant of Guillain-Barre syndrome – A case report

**DOI:** 10.1016/j.jaim.2025.101159

**Published:** 2025-06-27

**Authors:** Pankaj Yadav, Vaishali Kuchewar, Nikita Bhusari, Tanika Yadav

**Affiliations:** a Department of Kayachikitsa, Mahatma Gandhi Ayurved College Hospital & Research Centre, Salod (H.), Datta Meghe Institute of Higher Education & Research Centre, Wardha, Maharashtra, India; b Department of Kayachikitsa, Mahatma Gandhi Ayurved College Hospital & Research Centre, Salod (H.), Datta Meghe Institute of Higher Education & Research, Wardha, Maharashtra, India; c Department of Musculoskeletal Physiotherapy, Ravi Nair Physiotherapy College, Datta Meghe Institute of Higher Education & Research, Wardha, Maharashtra, India

**Keywords:** Guillain-Barre syndrome, *Sarvangavata,* physiotherapy, *Ayurveda intervention*, Case report

## Abstract

Guillain-Barre syndrome (GB Syndrome) is an acute inflammatory immune-mediated polyneuropathy having, progressive weakness, and pain in limbs. An extended illness can potentially be devastating due to respiratory problems and autonomic dysfunction. Despite the effectiveness of IV Ig and plasma exchange, most GBS patients develop severe weakness and have a prolonged disease course, typically with incomplete recovery. This is a case of a 51-year-old male patient diagnosed with Guillain-Barre syndrome (acute motor axonal neuropathy with Miller Fisher syndrome). GBS was correlated with *sarvangagata vata* and was treated by an integrated approach including Ayurveda treatment, Physiotherapy and some modern medication. There was significant improvement in motor functions and he recovered completely within 60 days. According to the case facts, it may be inferred that an integrated therapeutic approach may facilitate a speedy recovery in Guillain-Barre syndrome.

## 1 Introduction

Guillain-Barre Syndrome (GBS) is an acute, flaccid, neuromuscular paralysis in which the immune system attacks the peripheral nerves [1]. Although rare, with an incidence of 0.4–2 per 100,000, Guillain-Barré syndrome (GBS) has major effects on the healthcare system [2]. Males are affected at a slightly higher incidence than females [3]. GBS can be divided into different types based on clinical characteristics, etiology, and pathology. Acute motor-sensory axonal neuropathy (AMSAN), Acute inflammatory demyelinating polyneuropathy (AIDP), Acute motor axonal neuropathy (AMAN), and Miller Fisher syndrome (MFS) are the axonal variants of GBS [4]. It is often preceded by infection or other immune stimulation triggering an autoimmune response. Miller Fisher Syndrome (MFS) and Guillain-Barré Syndrome (GBS) are both rare, autoimmune disorders affecting the peripheral nervous system. Both MFS and GBS are characterized by an immune system attack on peripheral nerves, often triggered by preceding infections, which leads to demyelination and neurological symptoms [5]. The clinical manifestations and severity of the conditions diverge significantly. MFS is a variant of Guillain-Barré Syndrome and is primarily distinguished by a triad of symptoms: ophthalmoplegia, ataxia, and areflexia. Ophthalmoplegia refers to paralysis of the eye muscles, ataxia is the lack of muscle coordination, and areflexia denotes the absence of reflexes.Where as in GBS the predominant symptoms are gradual, legs are more commonly implicated than arms, giving the appearance of ascending paralysis progressing upwards, along with potential sensory disturbances and autonomic dysfunction [6]. The diagnosis is mostly based on clinical patterns. All GBS patients require close monitoring and assisting. From a diagnostic perspective, One of the key diagnostic tools for MFS is serological testing for anti-GQ1b antibodies. The presence of anti-GQ1b antibodies is strongly associated with MFS and is considered a biomarker for the condition. In contrast, GBS is associated with different antibodies, such as anti-GM1 and anti-GD1a, which are not typically seen in MFS. Cerebrospinal fluid (CSF) analysis is another important diagnostic procedure in both MFS and GBS. CSF often shows elevated protein levels with normal cell counts which helps to confirm the diagnosis by ruling out other causes of peripheral neuropathy.

In this patient clinical presentation of both GBS and MFS were present. Patient symptoms starts with ascending paralysis of lower limbs and progresses upwards involves paralysis of the eye muscles, lack of muscle coordination, and the absence of reflexes in lower limbs. Despite the effectiveness of IV Ig and plasma exchange, patients develop severe weakness and have a prolonged disease course, typically with incomplete recovery. Hence integrated approach is vital for fast recovery. This case was successfully treated with an integrated approach.

## 2 Patient information

A 51-year-old male patient was brought to the outpatient department of Kayachikitsa, in bedridden condition with complete immobility of both lower limbs, difficulty in chewing, unable to close eyes, slurred speech and Tingling Sensation all over the body. The patient had no history of hypertension and diabetes, or any prolonged ailment. He was a sweeper by profession and habitual of alcohol and tobacco for 20 years. On history taking, he was admitted in the neurology unit of the tertiary care center on 17/9/23 for complaints of sudden onset of giddiness, weakness in both lower limbs and slurred speech.

Patient investigations on 17/9/23 are mentioned in supplementary file 1 as table no S1.

Patient investigations on 17/9/23 was mentioned in supplementary file 1 as table no S1.

Management at Tertiary Care Centre (from 17-09-2023 to 26-09-2023) was mentioned in supplementary file 1 as table no S2.

On discharge from the tertiary care center, the patient was brought to Ayurveda Hospital on 7/10/23.

### 2.1 Clinical findings

The following were the findings of the examination on 7/10/23.

### 2.2 Timeline of treatment

The patient had taken treatments from September 17, 2023 to September 26, 2023 in tertiary care hospital (Table 1) and from October 07, 2023 to November 07, 2023 (Table 2) in our hospital.

**Table 1:** Examination findings of a patient on 7/10/23.

Sr. no	Clinical Examination	Clinical Examination findings
1	Vital parameters	• Blood pressure – 150/9 • Pulse – 118/min, regular • Respiration −23/min
2	**Neurological examination**	
2a	Mental status	• Patient was well-oriented and mentally stable
2b	Cranial nerve examination	• Oculomotor: Patient was unable to close the eyelids completely. • Trigeminal: Patient was having difficulty in mastication. • Facial: Patient was unable to close the eyelids completely, had difficulty in making facial expressions, ballooning of cheeks, and frowning of eyebrows. • Hypoglossal: Patient was unable to protrude and move the tongue. • The rest of the cranial nerves were normal.
2c	Motor examination	• **Muscle power** Right & left upper limb – Grade 4 Right & left lower limb - Grade 1
		• **Reflexes** – Superficial - abdominal & Planter – Absent Deep reflexes - Biceps & Triceps- Normal Knee Jerk & Ankle Jerk - Absent
2d	Sensory examination	• The following sensory parameters were normal Pain: Normal Light touch: Normal Temperature: Normal Position sense: Normal
3	Barthel Index [7] score	5
4	Hughes Functional Grading Scale [8] score	4
5	Other Systemic examinations	Within the normal limit

**Table 2:** Timeline of events.

Date and Event	Details
**Origin and Development**	
September 17, 2023 **(initial symptoms)**	• Sudden onset of giddiness, weakness in both lower limbs, slurred speech
September 17, 2023**(Diagnostic findings)**	• Complete blood count: Within normal limits • Kidney function test: Within normal limits • Random blood sugar: Within normal limits • Liver function test: Within normal limits • ECG: Normal sinus rhythm • Chest X-ray: No obvious abnormality • Nerve conduction study: Acute inflammatory demyelinating polyneuropathy
**Therapeutic interventions (modern)**	
September 17, 2023 - September 26, 2023 **(tertiary care hospital Management)**	• Inj Ceftriaxone (1 gm IV twice a day for 5 days) • Inj Thiamine (100mg IV thrice a day for 5 days) • Inj Dopamine D2S (200mg IV thrice a day for 5 days) • Inj Pantoprazole (40mg IV twice a day for 5 days) • Inj Ondansetron (4mg IV thrice a day for 5 days) • Inj Optineuron (vit B complex 3ml IV in 100ml NS once a day for 5 days) • Inj Tramadol Hydrochloride (50mg IV in 100ml NS thrice a day for 5 days) • Tab telmisartan (40mg twice a day) • Tab gabapentin & Nortriptyline (400mg twice a day for 10 days) • Syp Lactulose (15ml before sleep for 10 days)
**Progress and Further Development**	
October 07, 2023 **(admission to ayurveda Hospital)**	• Bedridden with complete immobility of both lower limbs • Difficulty in chewing, unable to close eyes, slurred speech, tingling sensation all over the body
October 07, 2023 **(clinical findings)**	• Blood pressure: 150/90 mmHg • Pulse: 118/min, regular • Respiration: 23/min • Mental status: Well-oriented and mentally stable • Cranial nerve examination: Involvement of Oculomotor, Trigeminal, Facial, and Hypoglossal nerves • Motor examination: Muscle power - Right & left upper limb (Grade 4), Right & left lower limb (Grade 1) • Reflexes: Superficial (abdominal & Plantar) - absent, Deep reflexes (Biceps & Triceps) - normal, Knee Jerk & Ankle Jerk - absent • Sensory examination: Normal for pain, light touch, temperature, and position sense • Barthel Index score: 5 • Hughes Functional Grading scale score:
**Therapeutic interventions (Ayurvedic)**	
October 07, 2023 - November 07, 2023 **(Ayurvedic procedures and Medications)**	• Shastikshali pinda sweda with Shatavari and Bala kwath (October 7, 2023 - November 06, 2023) for strengthening flaccid muscles • Shirodhara with brahmi taila (October 7, 2023 - October 21, 2023) for mental irritation and disturbed sleep • Akshi Tarpana with Triphala ghritam (October 7, 2023 - October 26, 2023) for strengthening eyelid movements • Nirgundi + shigru patra potli sweda at night (October 7, 2023 - November 06, 2023) for severe pain in both calf muscles • Nasya with ksheerbala taila (5 drops in each nostril) (October 7, 2023 - October 21, 2023) for urdhvajatrugata vata vikruti • Kala basti (Niruha basti with Dashmool + guduchi kwath and matra basti ashwagandha ghrita + ksheerbala tail) (October 7, 2023 - October 21, 2023) for shodhan and shaman of vitiated dosha • Mustadi Rajyapan basti (October 22, 2023 - November 05, 2023) for bruhan of mamsa & majja dhatu • Samshamni vati (250mg twice a day with warm water) as a rasayan (immuno-modulator), • Ekangveer rasa + Rasaraj rasa (125mg + 40mg twice a day with honey) for vatashaman • Tab shiv Gutika (250mg thrice a day with warm water) for muscle strengthening, • Dashmool kwath (20ml twice a day after meal with water) for analgesic properties • Saraswata ristha (20ml twice a day after meals with water) to improve mental functions, • Syp Amlycure Ds (20ml twice a day before meals with water) to improve appetite and hepatoprotection (chronic alcoholic)
**Follow-up and recovery**	
**1^ST^ follow-up on December 10, 2024**	• Assessment of vital parameters, including neurological function, and patient's overall well-being were noted normal. • The patient was well-oriented and mentally stable. Significant relief in symptoms was noted. The patient was now able to walk. Only tingling sensation was present on and off. All medications were stopped and only *Samshamni vati* 250mg BD was given for 2 months.
**2^ND^ follow up on March 5, 2024**	• No any further complaints. All medications were stopped and patient was now able to do his routine work like before.
**3^RD^ follow up on June 10, 2024**	• No recurrence of symptoms was noted and patient was doing good without any medications.
**4**th **follow up on July 15, 2024**	• No recurrence of symptoms was noted and patient was doing his routine job and work
**Telephonic follow up was done on August 27, 2024**	• Patient was maintaining good health without the need for any medications.
**5**th **follow up was on September 20, 2024**	• Patient doing his regular work efficiently without the need of any medications.

Here is the detailed timeline table based on the patient's case, including diagnostic, therapeutic (modern and Ayurvedic treatments), and follow-up are given in Table 2.

### 2.3 Therapeutic intervention

The treatment was planned considering the predominance of vata dosha and the involvement of *mamsa* and *majja dhatu*. At the time of admission patient was on antihypertensive medicine (Telmisartan 40 mg twice a day) and on Tab Gabapentin & Nortriptyline 400mg twice a day.

Ingredients of *Basti* (medications by rectal route) was mentioned in supplementary file 1 as table no S3.

With the above mentioned ayurveda intervention (Table 2) physiotherapy was also advised as per Table 3.

**Table 3:** Physiotherapy done during hospital stay.

Physiotherapy			
S.No.	Details of Physiotherapy	Duration	Goal of the treatment.
1	Passive movements	7-10-2023 to 13-10-2023	To maintain joint mobility
2	Positional Shifting for	7-10-2023 to 13-10-2023	To prevent pressure sores
3	TENS (Transcutaneous electrical nerve stimulation)	7-10-2023 to 20-10-2023	To stimulate sensory nerves and subsequently activate the opioid system and/or the pain gate mechanism in order to relieve symptomatic pain
4	PNF (Proprioceptive neuromuscular facilitation	7-10-2023 to 20-10-2023	To promote improved motor performance and control.
5	Abdominal strengthening exercises	12-10-2023 to 30-10-2023	To increase the strength of abdominal muscles.
6	Isometric and isotonic exercises	12-10-2023 to 30-10-2023	To improve strength, stability and functional ability.
7	Training on parallel bar gait	15-10-2023 to 30-10-2023	To establish proper motor patterns involved in walking.

Initially patient had a burning sensation in both lower limbs, a lack of appetite and constipation, hence kala basti was planned for *shodhan* and *shaman* of vitiated dosha. After *shodhan* and the improvement of *agni, bruhan basti(mustyadi Rajyapan basti)* was administered. In *shaman Chikitsa*, *Vatashamak, agnivardhak, mamsa* and *majja dhatuposhak, rasayan* medicines were selected (Table 4).

**Table 4:** Treatment on discharge.

S. No	Medicine	Dose & duration	Time for administration	Adjuvant	Duration
1	*Balaristha*	20ml twice a day	After meal	Warm water	15 days
2	*Ekangveer rasa + Rasaraj rasa*	125mg + 40mg twice a day	After meal	With Honey	15 days
3	*Aswagandha ghrita*	10ml once a day	Empty stomach	Warm water	30 days
4	*Samshamni vati*	250mg once a day	After meal	Warm water	30 days
5.	*Shiv Gutika*	250 mg thrice a day	After meal	Warm water	30 days
6.	Syp Amlycure DS	15ml twice a day	After meal	Warm water	30 days

### 2.4 Observation

As the treatment was started, the patient's blood pressure decreased, hence the dose of telmisartan was reduced to 40 mg once a day from October 16, 2023 and gabapentin was discontinued with tapering dose. Patient completely recovered and resumed his duty from January 20, 2024 (Table 5).

**Table 5:** Pre and post-treatment assessment of patient 'clinical features', 'Vitals', and 'Neurological assessment'.

Sr. no.	Observations	Before Treatment (October 07, 2023)	After Treatment (07/112024)
1	Blood pressure (in mm of Hg)	150/90	130/80
2	Pulse Rate (in beats/minute)	118	78
3	Respiration rate (per minute)	23	20
4	Barthel Index (Total score from 0 to 20, with lower score indicating increased disability)	05	18
5	Hughes Functional Grading scale (a measure of disability to rate clinical performance ranging from 0 to 6 with higher numbers indicating more severe disability).	04	01
6	Power grading ➢ Right upper limb ➢ Left upper limb ➢ Right lower limb ➢ Left lower limb		
		04	05
		04	05
		01	04
		01	04
7	Reflexes **Deep tendon reflexes** Biceps reflex Triceps reflex Knee Jerk reflex Ankle Jerk reflex **Superficial tendon reflexes** Abdominal reflex Plantar reflex	Normal Normal Absent absent Absent absent	Normal Normal Normal Normal Normal Normal
8	Complete immobility of both lower limbs	Normal movement of both lower limbs	
9	Difficulty in chewing	Normal chewing	
10	Watering of eyes	Mild watery discharge	
11	Unable to close eyes	Normal movements of eyelids	
12	Slurred speech	Normal speech	
13	Tingling sensation all over body	Mild tingling sensation in both lower limbs on/off.	

## 3 Discussion

This patient had no history of bacterial or viral infection which is one of the etiological factors of the GBS. He had regular consumption of alcohol and tobacco for years which might be the associated factor. Based on clinical presentation, it can be correlated with *sarvangagata Vata.* The treatment comprised of *abhyantar* and *bahya chikitsa* (internal and local treatment) along with regular sessions of physiotherapy.

The patient was suffering from constipation, kala basti was started with Niruha basti of *dashmoola* and *guduchi kwath* and *matra basti* of *ashawagandha ghrita* and *ksheerabala taila for dosha Shodhana and shaman respectively.* Later on, *Mustayadi rajyapana basti* was administered to provide nourishment to *mamsa* and *majja dhatu* [9].

*Shastika shali pinda sweda* was done to give strength to muscles, as the calf muscles were flaccid. The study conducted by A Vyas et al. also found improvement in muscle power and tone with *shashtikshali pinda sweda* [10]. *Akshi Tarpana* (therapeutic retention of medicated liquids over the eyes) with *Triphala Ghritam* [11] showed significant improvement in closing the eyelids completely. The study conducted by GM Timmapur et al. showed effect of *Triphala ghritam* for nourishment and strengthening of ocular structures thus providing relaxation to the eyes, improving the functioning capacity of the eyes, and helping in the closing and opening of eyelids completely. Local *nirgundi* and *shigru patra potali swedan* was done at night to reduce the pain in lumbar region and calf muscles. *Nirgundi* and *shigru* acts as *shoolhara* and *vatashaman* thereby reducing pain [12] *Shirodhara* with *brahmi taila* showed significant improvement in tachycardia, and hypertension. One Study conducted by Dhuri KD et al., showed that *Shirodhara* induce bradycardia and lowers sympathetic tone. There was a significant improvement in mood scores and the level of stress [13]. *Nasya* with *ksheerbala taila* (medicated oil) is indicated in the management of *Vataja* disorders [14]*. Ksheerbala taila* contains lipid-soluble compounds that have a higher propensity to passively absorb through the nasal mucosa and crossing the blood-brain barrier (BBB). The glycosides present in the *Sida cordifolia* has confirmed to exert anxiolytic, nerve strengthening and sedative effects on the central nervous system.

Following medicinal preparations were used considering their various properties.

***Balaristha-*** The key ingredients are *Bala* and *Ashwagandha* which are mentioned as *Vatahara* and indicated in *Vatavyadhi*, It is *Balya*, *Brimhana* (nourishing) and *Agni vardhak* (enhance digestion & metabolism [15

***Sanshamani vati*** contains Guduchi (Tinospora cordifolia [Wild.] Miers.) which is one of the Rasayana (Rejuvenating) drugs mentioned in Ayurveda. It is an anti-inflammatory, antioxidant effect [16].

***Shivagutika*** contains *Shilajatu* (Asphaltum Punjabianum) as a key ingredient It is a rich source of bioactive components. It increases the uptake of minerals like calcium, magnesium, and phosphate into the tissues of muscles and strengthen them. It is a Rasayana and helpful in *Vata* and *Shiroroga* [17].

***Saraswataristha*** contains *Brahmi* (Bacopa monnieri), *Shankhpushpi* (Convolvulus pluricaulis), *Ashwagandha* (Withania somnifera). It possesses neuroprotective properties. It is primarily known for promoting overall mental health [18].

***Rasaraj rasa*** contains *suvarn bhasma*, *shuddha vishamushti, ashwagandha, rasasindoor, guggul,* and other ingredients. It is indicated in all types of *Vatajvikara* (diseases due to *Vata dosha*). Its anti-inflammatory and antioxidant qualities protect the body from oxidative damage and free radicals [19].

*Ekangaveera Rasa* is effective in *Vataja* disorders. which is useful in imparting strength to muscle fibers. It aids in lowering muscle pain, stiffness, swelling and inflammation in the muscles [20].

***Dashmool kwath*** a combination of *Brihat Panchamoola* and *Laghu Panchamoola.* It alleviates *vata Dosha* and reduces its aggravation. It has potent anti-inflammatory, antioxidant, and analgesic properties. It cures a variety of diseases including nerves, muscles, bones, and joints [21].

**Amlycure DS –** It mainly contains, *kutaki, Kalmegh, Sharpunkha, Tulsi, Bhumyamalaki*, and *Punarnava.* It helps to maintain the integrity of hepatocytes. It improves appetite, digestion, and protects the liver from toxins & boosts immunity.

***Aswagandha ghrita*** is specifically indicated in *Vataroga* [22]. having rejuvenating and adaptogenic properties. The study conducted by NJ Dar et al. found that it has lipophilic activity, which enables it to pass through cell membranes, and natural steroids, which improve protein synthesis [23]. *Ashwagandha ghrita* contains fatty acids and phospholipids which promote protein synthesis. This may have been responsible for the nerve strengthening.

**Physiotherapy-** During the initial phase, passive movements were performed throughout their entire range of motion to maintain joint mobility. To prevent pressure sores, a 2-h shift in the patient's position from supine to side-lying was administered because the patient was immobile during the first few days.

In addition, he reported experiencing severe pain from lumber to both lower limbs, which was managed with TENS (Transcutaneous electrical nerve stimulation); which primarily seeks to stimulate sensory nerves and subsequently activate the opioid system and/or the pain gate mechanism to relieve symptomatic pain. Patients received regular stretching of the hamstrings, piriformis, achilles tendon, to prevent muscle tightness.

**Proprioceptive neuromuscular facilitation** (PNF) was performed by assisted facilitation of a weak group of muscles & resisted which is strong muscles with reciprocal inhibition patterns to promote improved motor performance and control. Stimulating the nerves and enhancing motor performance is crucial because lower and upper limb weakness is caused by decreased nerve transmission. Therefore, the purpose of the electrical stimulation was to improve muscular performance. To reduce early tiredness and improve muscle function, energy conservation and compensation measures were designed. The patient was also instructed on how to use their extremities to participate in daily tasks. As the progression was seen in achieving a full range of motion, the strengthening of muscles was started with TheraBand, dumbbell and weight cuff. Individual muscles of the upper and lower limbs were targeted. The patient was also having weak abdominal strength, so abdominal strengthening exercises like bridging and static curl-ups were practiced. Isometric and isotonic exercises were administered to the patient as they made progress to strengthen their muscles, while endurance training entailed progressively increasing the time and intensity of physical activities like walking or climbing stairs. Later on; to strengthen the hip flexor, extensor, and lateral rotator; training on parallel bar gait was provided by performing hip hiking, leg swinging, one leg standing.

The findings of this case suggests that Ayurveda intervention with physiotherapy helps in fast recovery in acute motor axonal neuropathy with Miller-Fisher syndrome.

## 4 Patient perspective (December 10, 2024)

I was bedridden, had severe pain in both lower limbs and was unable to speak. I was able to walk with support within 1 month and improvement in other complaints also. I thus grant permission for my case to be used in scientific research.

## 5 Informed consent

The patient provided informed consent to be published.

## Author contributions

Treated the case-VK; Conceptualization-VK, PY; Drafting the article: VK, PY; Analyzing the medical intervention-VK, PY; Revising it critically-NB, TY; Methodology.; NB, PY; Writing - Review & Editing; NB, TY; Final approval of the version to be submitted-VK, PY.

## Funding sources

None.

## Declaration of generative AI in scientific writing

We state that any help from generative AI or AI assisted technology has not been obtained in writing of this manuscript.

## Declaration of competing interest

The authors declare that they have no known competing financial interests or personal relationships that could have appeared to influence the work reported in this paper.
