# Supplementary material for: Integrated treatment approach to Miller Fisher syndrome, a variant of Guillain-Barre syndrome – A case report
Source: J Ayurveda Integr Med. 2025 Jun 27;16(4):101159. doi: 10.1016/j.jaim.2025.101159 (PMC12268623; doi:10.1016/j.jaim.2025.101159)
Supplement: Multimedia component 1 [file mmc1.docx]

**A note on medicinal preparations and their properties**

**Introduction to Medicinal Plants in the Case Study**

In this session, we will explore the medicinal plants used in treating a 51-year-old male patient diagnosed with Acute Inflammatory Demyelinating Polyneuropathy (AIDP). The patient experienced symptoms such as immobility, difficulty chewing, inability to close his eyes, slurred speech, and tingling sensations. Ayurvedic treatments were integrated with modern medical interventions to address these issues. The focus will be on the therapeutic properties and applications of the medicinal plants involved in the treatment**.**

**Key Medicinal Plants Used in the Treatment**

***Ashwagandha*** (Withania somnifera)

Properties: Ashwagandha is known for its adaptogenic, anti-inflammatory, antioxidant, and neuroprotective properties.

Applications: It was used in Ashwagandha ghrita and other formulations to strengthen nerves, reduce inflammation, and support overall vitality.

***Bala* (Sida cordifolia)**

Properties: *Bala* is recognized for its *Vatahara* (pacifying Vata), *Balya* (strengthening), and *Brimhana* (nourishing) properties.

Applications: Included in *Balaristha*, Bala helps in regenerating and strengthening muscles, aiding in the recovery of muscle tone and strength.

***Guduchi* (Tinospora cordifolia)**

Properties: *Guduchi* is a *Rasayana* (rejuvenative) with anti-inflammatory and immunomodulatory effects.

Applications: Present in *Sanshamani vati, Guduchi* supports the immune system and helps reduce inflammation, aiding in the body's recovery process.

***Triphala***

Components: Comprising *Haritaki (*Terminalia chebula), *Bibhitaki* (Terminalia bellirica), and *Amalaki* (Emblica officinalis).

Properties: *Triphala* has antioxidant, digestive, and rejuvenative properties.

Applications: Used in *Triphala Ghritam* for Akshi Tarpana, it enhances ocular health and improves eyelid function.

***Shatavari* (Asparagus racemosus)**

Properties: *Shatavari* is known for being adaptogenic, nourishing, and cooling.

Applications: Part of *Shastikshali pinda sweda* with *Shatavari* and *Bala kwath,* it helps nourish and strengthen muscles.

***Nirgundi* (Vitex negundo)**

Properties: *Nirgundi* is analgesic, anti-inflammatory, and *Vatahara.*

Applications: Used in *Nirgundi* and *Shigru patra potli sweda,* it alleviates pain in the lumbar region and calf muscles.

***Shigru* (Moringa oleifera)**

Properties: *Shigru* has anti-inflammatory, analgesic, and antioxidant properties.

Applications: Combined with *Nirgundi* in *potli sweda,* it provides pain relief and reduces inflammation.

***Brahmi* (Bacopa monnieri)**

Properties: *Brahmi* is neuroprotective, enhances cognitive function, and has anxiolytic properties.

Applications: Utilized in *Saraswataristha* and *Shirodhara* with *Brahmi taila,* it improves mental functions, reduces stress, and supports cognitive health.

***Dashmoola***

Components: A combination of ten roots, including *Bilva* (Aegle marmelos), *Agnimantha* (Premna integrifolia), *Shyonaka* (Oroxylum indicum), *Patala* (Stereospermum suaveolens), *Gambhari* (Gmelina arborea), *Brihati* (Solanum indicum), *Kantakari* (Solanum xanthocarpum), *Shalaparni* (Desmodium gangeticum), *Prishniparni* (Uraria picta) and *Gokshura* (Tribulus terrestris) - Used for treating joint disorders and as a muscle relaxant.

Properties: *Dashmoola* is anti-inflammatory, analgesic, and *Vatahara.*

Applications: Used in *Dashmool kwath* and *Niruha basti,* it alleviates inflammation and balances *Vata dosha*.

**Discussion on the Therapeutic Integration**

Internal and External Therapies

**Internal:** Medicines such as *Balaristha, Sanshamani vati, Shivagutika, Saraswataristha,* and *Rasaraj rasa* were administered to nourish and strengthen the nervous system, reduce inflammation, and support overall health.

**External:** Procedures like *Shastikshali pinda sweda*, *Akshi Tarpana, Nirgundi + Shigru patra potli sweda,* and *Shirodhara* with *Brahmi taila* were employed to target specific symptoms such as muscle weakness, pain, and ocular issues.

**Rationale for Selection**

The selection of these plants and formulations was based on their documented properties and their ability to address the specific symptoms and underlying pathophysiology of AIDP and *Vata* disorders in Ayurveda. *Shodhana* (purification) and *Shamana* (pacification) therapies were used to balance the *doshas,* particularly *Vata*, which is crucial in neurodegenerative conditions.
