# Supplementary material for: Integrated treatment approach to Miller Fisher syndrome, a variant of Guillain-Barre syndrome – A case report
Source: J Ayurveda Integr Med. 2025 Jun 27;16(4):101159. doi: 10.1016/j.jaim.2025.101159 (PMC12268623; doi:10.1016/j.jaim.2025.101159)
Supplement: Multimedia component 2 [file mmc2.pdf]

Integrated treatment approach to Miller Fisher syndrome, a variant of Guillain-Barre Syndrome – A Case report

S 1: Patient investigations on 17/9/23

Sr. no	Investigations findings
1.	Complete blood count- within normal limit
2.	Kidney function test - within normal limit
3.	Random blood sugar - within normal limit
4.	Liver function test - within normal limit
5.	ECG shows normal sinus rhythm.
6.	Chest X ray suggests No Obvious Abnormality.
7.	Nerve Conduction Study Showed Acute Inflammatory Demyelinating Polyneuropathy.

S 2: Management at Tertiary Care Centre (from 17-09-2023 to 26-09-2023)

**Sr. No.**	**Intervention**	**Dose & Duration**
1	Inj Ceftriaxone	1gm IV twice a day for 5 Days
2	Inj Thiamine	100 mg IV thrice a day for 5 Day
3	Inj Dopamine D2S	200mg IV thrice a day for 5 Days
4	Inj Pantoprazole	40mg IV twice a day for 5 Days
5	Inj Ondansetron	4mg IV thrice a day for 5 Days
6	Inj Optineuron (vit B complex)	3ml Iv in 100ml NS once a day for 5 Days
7	Inj Tramadol Hydrochloride	50 mg Iv in 100ml NS thrice a day for 5 Days
8	Tab Telmisartan	40mg twice a day
9	Tab Gabapentin & Nortriptyline	400 mg twice a day for 10 Days
10	Syp Lactulose	15 ml before sleep for 10 Days

S 3: Ingredients of *Basti* (medications by rectal route)

Sr. no.	Type of Basti	Ingredients
1	*1*Anuvasana basti*	*Ashwagandhaghrita(40ml)+ Ksheerbala tail (40ml)*
2	*1*Niruha basti*	*Dashmool+ Guduchi kwath* - 400ml<br>*Madhu* - 40ml<br>*Sainadhava Lavana* - 10gm<br>*balaashwagandha tail* -50ml<br>*Guduchi Churna kalka Dravya*- 10gm
3	2* *Mustadi Rajyapan basti*	*Mustabharad kwath* - 100ml<br>*Madhu*- 40ml<br>*Sainadhava Lavana* -10gm<br>*balaashwagandha tail* -50ml<br>*Guduchi Churna kalka* - 10gm<br>*Majja (meat soup)*- 100ml
